# Facilitating telemedicine project sustainability in medically underserved areas: a healthcare provider participant perspective

**DOI:** 10.1186/s12913-016-1401-y

**Published:** 2016-04-26

**Authors:** David L. Paul, Reuben R. McDaniel

**Affiliations:** Department of Business Information and Analytics, Daniels College of Business, University of Denver, Denver, Colorado USA; McCombs School of Business, The University of Texas at Austin, Austin, Texas USA

**Keywords:** Telemedicine, Telehealth, Medically underserved areas, Sustainability

## Abstract

**Background:**

Very few telemedicine projects in medically underserved areas have been sustained over time. This research furthers understanding of telemedicine service sustainability by examining teleconsultation projects from the perspective of healthcare providers. Drivers influencing healthcare providers’ continued participation in teleconsultation projects and how projects can be designed to effectively and efficiently address these drivers is examined.

**Methods:**

Case studies of fourteen teleconsultation projects that were part of two health sciences center (HSC) based telemedicine networks was utilized. Semi-structured interviews of 60 key informants (clinicians, administrators, and IT professionals) involved in teleconsultation projects were the primary data collection method.

**Results:**

Two key drivers influenced providers’ continued participation. First was severe time constraints. Second was remote site healthcare providers’ (RSHCPs) sense of professional isolation. Two design steps to address these were identified. One involved implementing relatively simple technology and process solutions to make participation convenient. The more critical and difficult design step focused on designing teleconsultation projects for collaborative, active learning. This learning empowered participating RSHCPs by leveraging HSC specialists’ expertise.

**Conclusions:**

In order to increase sustainability the fundamental purpose of teleconsultation projects needs to be re-conceptualized. Doing so requires HSC specialists and RSHCPs to assume new roles and highlights the importance of trust. By implementing these design steps, healthcare delivery in medically underserved areas can be positively impacted.

## Background

Telemedicine, “the use of electronic information and communications technologies to provide and support healthcare when distance separates the participants” ([1] p. 2), is perceived as enabling improvements in healthcare delivery and outcomes [2]—particularly in areas and populations where healthcare resources are lacking or unavailable [3–5]. Telemedicine and telehealth both describe the use of healthcare information exchanged from one site to another via information and communications technologies to improve the patient’s health status. This research focuses on a particular type of telemedicine, teleconsultations, which are consultations between two or more geographically separated healthcare providers connected through information and communications technologies to provide value-added healthcare delivery [1, 6, 7]. Teleconsultations generally can be between a primary care provider (family practice physician, nurse practitioner, or physician assistant) located at a local hospital or clinic and the relevant specialist(s) located at a university-affiliated health sciences center.

Access to both primary and specialty care is a major challenge throughout many parts of the world. In the United States alone, more than 4,000 areas and populations are classified as medically underserved [8]. More than 55,000,000 people live in the 5,766 designated Health Professional Shortage Areas (HPSAs) [9], with 77 % of the nation’s 2,050 rural counties designated as HPSAs [10]. This situation is likely to deteriorate further because the shortage of primary care physicians is estimated to increase from approximately 39,000 in 2015 [11] to 124,000 by 2025 [6].

Telemedicine is perceived as an effective and efficient means by which to partially address such challenges [12–14]. For example, the Patient Protection and Affordable Care Act of 2010 specifically identifies telehealth as an innovative means by which to provide and coordinate care related to chronic conditions and behavioral health issues for medically underserved areas, and as a meaningful tool for accountable care organizations to provide high quality and efficient healthcare services in a cost effective manner [15, 16].

### The challenges of sustainability

The early utilization rates of installed telemedicine projects in the 1990s were disappointingly low [15, 17]. Since then, the efficacy and efficiency of numerous types of clinical applications in teleconsultations have been demonstrated [4, 18, 19]; significant financial support has been given to telemedicine projects targeting medically underserved areas such as rural America [20, 21]; the capabilities, usability, and affordability of telemedicine technology have increased [22]; studies have demonstrated how telemedicine projects can be successfully implemented [23–25]; and numerous telemedicine projects have been successfully implemented [18, 26]. Despite this, telemedicine utilization rates have remained low [26], and very few of the implemented telemedicine projects have been sustained over time—despite a continued need for the services provided [23, 25, 27].

Sustainability, the ability of a telehealth service to continue functioning into the future in terms of adding a level of telehealth activity into an existing clinical setting or reaching a critical mass on its own [28], is a complex multifaceted phenomenon that has been perceived as the “holy grail of telehealth” ([29] p. S7). Prior research on telemedicine service sustainability includes a multifactor meso-level model [30] based on Normalization Process Theory [31, 32]. Clinician acceptance was identified as the key factor. Other factors were clinician workforce availability, adequate technology, telehealth champions, positive beliefs about telehealth, good relationships between providers, clinician demand for services, and resourcing [30]. Many of these factors affect and highlight the importance of healthcare providers’ role in determining teleconsultation project sustainability. Thus suggests that a way to better understand telemedicine service sustainability is to 1) focus on teleconsultation projects that have been successfully implemented, and 2) examine them from the perspective of the participating healthcare providers. In terms of clinician acceptance, there are certain characteristics of teleconsultation session goals, activities, and processes that differ between sustained and dormant projects. Clinician workforce availability is an important factor. Given current and forecasted healthcare professional shortages, how do organizations support clinician participation in a manner that does not require the hiring of additional clinicians? Further, adequate technology is also a factor, but are there commonalities in what is considered adequate and how does that relate to the purpose and process of teleconsultation sessions?

This research builds on and extends prior research by developing a better understanding of telemedicine service sustainability in order to identify the critical drivers influencing healthcare professionals’ continued participation, and how projects can be designed to address these challenges. The following research questions are addressed:

1. What are the significant drivers influencing healthcare providers’ continued participation in teleconsultation projects?
2. How can teleconsultation projects be designed to effectively and efficiently address these drivers?

## Methods

### Research design

Case studies of fourteen teleconsultation projects from two active telemedicine networks (Sites Y and Z) were studied. Comparative case studies were utilized to research teleconsultations because case studies are an appropriate methodology when studying contemporary phenomenon occurring within a real-life context [33–35]. Further, teleconsultations are complex adaptive systems [36], and case studies are also an appropriate methodology when studying such systems – especially in healthcare [37]. The advantages of utilizing case studies in such circumstances is that they can increase the robustness and generalizability of the findings through replication and the use of multiple sources of evidence [33–35]. Data from Site Y were collected at two points in time nearly a decade apart while data from Site Z were collected at the later data collection period only. The Institutional Review Board (IRB) of the university approved these projects.

### Sample

Telemedicine networks, consisting of a university-affiliated health sciences center (HSC) as the hub and smaller healthcare facilities as the spokes, were purposely selected because the vast majority of civilian telemedicine projects involve HSCs [1]. HSC telemedicine networks tend to have certain characteristics that naturally account for alternative explanations of installed telemedicine project utilization [38]. Three telemedicine networks (Sites W, X, and Y) were initially studied in the first data collection period. The major selection criteria was that they had to have active telemedicine projects that included teleradiology and distance learning in addition to teleconsultation projects (see Paul and McDaniel [39] for a detailed description of site selection criteria and process).

The researcher planned to revisit these sites in order to study how these telemedicine networks, and in particular their teleconsultation projects, had evolved. Unfortunately, both Sites W and X had decided to discontinue or deemphasize their teleconsultation efforts, and the teleconsultation projects previously studied had been discontinued. Site X had decided to focus on distance learning only, and Site W had significantly deemphasized their teleconsultation efforts because state funding for the HSC as a whole had been significantly reduced, and their teleconsultation projects were one of many efforts whose funding was cut.

The second data collection period included data from Sites Y and Z. Site Y had expanded their teleconsultation project efforts, and Site Z, which did not have any active projects during the first data collection period, was included because it had deployed a number of different teleconsultation projects and its inclusion enabled between telemedicine network comparisons.

Table 1 presents background and demographic information about Sites Y and Z. A total of fourteen teleconsultation projects in twelve geographical locations were studied (two remote areas had two different teleconsultation projects located in the area). All of the remote sites were designated as either medically underserved areas or populations, and twelve of the fourteen remote sites were designated as primary care HPSAs. The two remote sites not designated HSPAs, ZB and ZC, were located in the same relatively isolated city and surrounded by areas within the county that were designated HPSAs.

**Table 1:** Teleconsultation projects background and demographics

HSC	Project	Data Collection Period	Projects Located in Same Areas	Teleconsultation Activites	Census Classification	Population	MUA/MUP	HPSA-Primary Care	Distance from HSC (miles)	Project Tenure (A Time of Data Collection)	Additional Data Sources
Y	Y1	Both		Multiple Medical Specialties	Nonmetropolitan	Period 1 8,700 Period 2: 9,300	MUA (Entire County)	Population Group Low Income	400 (200 to Affiliated HSC	4 years (1997) 14 years (2007)	A, B
	Y2	1		Multiple Medical Specialties	Nonmetropolitan	Period 1: 6,500 Period 2: 7,200	MUA (Entire County)	Entire County	460 (250 to Affiliated HSC	3 years (1997)	
	Y3	2		Burn Unit	Metropolitan	680,000	MUA (Partial--Multiple Service Areas)	Facility and Service Area	300	4 years	
	Y4	2		Oncology	Nonmetropolitan	7,700	MUA (Entire County)	Entire County	145 (120 to affiliated HSC)	4 months	
	Y5	2		Primary Care	Nonmetropolitan	1,100	MUA (Entire County)	Entire County	80 (50 from Remote PCP)	6 years	F
	Y6	2		Pediatric Care	Nonmetropolitan	8,200	MUA (Entire County)	Entire County	60	9 years	F
Z	Z1	2	Z7	Hepatitis C (HCV)	Metropolitan	52,000	MUA (Entire County)	Entire County	275	2 years	B, C, D, E
	Z2	2	Z3	Hepatitis C (HCV)	Metropolitan	174,000	Governor’s MUP	No (Adjacent areas within countyare)	320	3 years	B, C, D, E
	Z3	2	Z2	Hepatitis C (HCV)	Metropolitan	174,000	Governor’s MUP	No (Adjacent areas within countyare)	320	2 years total (current RSHCP 6 months)	B, C, D, E
	Z4	2		Hepatitis C (HCV)	Nonmetropolitan	41,000	MUA (Entire County)	Facility and Service Area	90	3 years	B, C, D
	Z5	2		Early Childhood Developmental Disabilities (ECDD)	Nonmetropolitan	6,000	MUA (Entire County)	Population Group: Low Income	280	5 years	B
	Z6	2		Early Childhood Developmental Disabilities (ECDD)	Nonmetropolitan	25,000	MUA (Entire County)	Population Group: Low Income	230	2 years	B
	Z7	2	Z1	Early Childhood Developmental Disabilities (ECDD)	Nonmetropolitan	52,000	MUA (Entire County)	Entire County and Facility	275	2 years	B
	Z8	2		Drug Abuse & Behavioral Counseling (DABC)	Nonmetropolitan	26,000	MUA (Entire County)	Facility	60	6 months	

A Teleconsultation Session Videorecording

B HSC Internal Documents

C HSC Public Documents

D HSC Web Sire

E Session Observation (from HSC)

F Remote Site Internal Documents

Population size of the remote sites varied. Eleven of the fourteen remote sites were in areas located in US Department of Health and Human Services designated non-metropolitan (population less than 50,000), with the rest being defined as metropolitan (population over 50,000), and the ratio of sites studied that were classified as metropolitan or nonmetropolitan (33 %/66 %) is consistent with the United States as a whole (27 %/73 %) [40]. Each remote site was relatively isolated geographically, with the nearest HSC being a minimum of 60 miles away. Nine of the fourteen projects remote sites were located 200 or more miles from the nearest HSC.

Table 2 presents an overview of the teleconsultation projects themselves. Site Y had two multiple medical specialties teleconsultation projects (Y1 and Y2) active at the time of the first data collection period. Five teleconsultation projects, including Y1 from the first period (Y2 had been discontinued) were part of the second data collection period. The four additional teleconsultation projects included a burn unit, oncology, primary care (where the teleconsultation project involved a remote site primary care physician linked to an even smaller town which also had a telepharmacy link with the HSC), and pediatric care at a school clinic. Site Z had eight teleconsultation projects involving three different clinical applications from which data were collected. These included teleconsultation projects focused on treating hepatitis C (Project HCV), early childhood developmental disabilities (Project ECDD), and drug abuse and behavioral counseling (Project DABC).

**Table 2:** Teleconsultation projects site overview

HSC	Project	Data Collection Period	Teleconsultation Activities	Main HSC Participants	Remote Facility	Remote Care Provider	Information Technology Configuration	Utilization (approx.)
Y	Y1	Both	Multiple Medical Specialties	Multiple Specialists	Hospital	Primary Care Physicians	Specially Designed	Period 1: 8 times per month^a^ Period 2: 1 time per month
	Y2	2	Multiple Medical Specialties	Multiple Specialists	Clinic	Physician Assistant (PA)	Telemedicine Workstation	Period 1: <1 time per month^b^
	Y3	2	Burn Unit	Subspecialist	Hospital	Nurse	(using off-the-shelf components) with	1 h every other week
	Y4	2	Oncology	Subspecialist	Hospital	Primary Care Physicians		
	Y5	2	Primary Care	Primary Care Physician at Remote Clinic^d^	Other Remote Clinic	Emergerncy Medical Technician (EMT)	Videoconferencing +	Multiple times per week
	Y6	2	Pediatric Care	Multiple Specialists	Clinic	Nurse	Attachments	2 h per week + as needed
Z	Z1	2	Hepatitis C (HCV)	Specialists, Subspecialists, Therapists, Nurse, Practitioners, Nurse, Counselors	Hospital	Primary Care Physician	Multipoint Teleconferencing	2 h per week + 2 h behavioral per week (total)
	Z2	2	Hepatitis C (HCV)	Specialists, Subspecialists, Therapists, Nurse, Practitioners, Nurse, Counselors	Hospital	Resident and Nurse	Multipoint Videoconferencing	2 h per week + 2 h behavioral per week (total)
	Z3	2	Hepatitis C (HCV)	Specialists, Subspecialists, Therapists, Nurse, Practitioners, Nurse, Counselors	Hospital	Internist	Multipoint Teleconferencing	2 h per week + 2 h behavioral per week (total)
	Z4	2	Hepatitis C (HCV)	Specialists, Subspecialists, Therapists, Nurse, Practitioners, Nurse, Counselors	Clinic	Physician Assistant	Multipoint Videoconferencing	2 h per week + 2 h behavioral per week (total)
	Z5	2	Early Childhood Developmental Disabilities (ECDD)	Specialist, Therapists, Counselors	Clinic	Developmental Specialist	Videoconferencing	3 h monthly (consults only)
	Z6	2	Early Childhood Developmental Disabilities (ECDD)	Specialist, Therapists, Counselors	Clinic	Developmental Specialist	Videoconferencing	3 h monthly (consults only)
	Z7	2	Early Childhood Developmental Disabilities (ECDD)	Specialist, Therapists, Counselors	Clinic	Developmental Specialist	Videoconferencing	3 h monthly (consults only)
	Z8	2	Drug Abuse and Behavioral Counseling (DABC)	Psychiatrist	In-patient Clinic	Psychologists, Mental Health Counselor	Videoconferencing	2 h per week (total)^c^

^a^Y1 rural hospital expanded and now has many specialists

^b^Y2 was discontinued when PA moved and supervising physician died

^c^At time of data collection, Project DABC was rolling out additional new sites in the near future

^d^Y5 had a telepharmacy project with HSC Y, and a teleconsultation project with a primary care physician located approximately 60 miles from both Y5 and HSC Y

### Data collection

Data were collected at two points in time (1996/1997 and 2007) approximately 10 years apart, and the primary data collection method involved face-to-face, issue-focused, semi-structured interviews of key informants. The time elapsed between the two data collection periods was based on a desire to be sure that projects had been in existence long enough to become institutionalized in the delivery setting. Face-to-face interviews were required to collect the thick and richly textured data that were needed to understand the topics being researched [41, 42] because, prior to the first data collection period, telephone interviews were pretested and found ineffective.

Table 3 presents an overview of the distribution of key informants, who were members of one of three groups—clinicians (physicians, physician assistants, nurse practitioners, medical residents, nurses, or, in one case, an emergency medical technician), administrators, and IT professionals. They were selected based on current or past direct involvement in their organization’s teleconsultation projects. A total of 60 healthcare professionals, 35 at Site Y and 25 at Site Z, were interviewed face-to-face, and the interviews were audiorecorded and transcribed. At Site Y, 17 were interviewed as during the first data collection period, whereas 21 (including three from the first period) were interviewed during the second data collection period. Signed informed consent forms informing the participants of their rights and stating that all participation was voluntary were obtained at the time of the interview from all key informants.

**Table 3:**
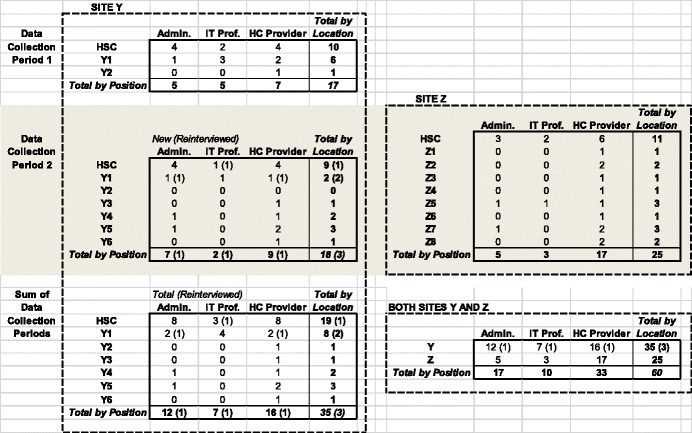
Key informants overview

Triangulated data collection in the form of obtaining different perspectives of teleconsultation projects was done by interviewing multiple key informants from the three different functional groups at both the remote healthcare facility (if multiple key informants existed) and the HSC involved in each teleconsultation project studied, and by collecting additional data types from sources that varied by teleconsultation project. As indicated in Table 1, additional data included observations of teleconsultation sessions or videorecordings of such sessions and documentation such as grant proposals/follow-up, needs assessments, and strategic plans. Both these data triangulation steps were taken in an effort to gain a richer understanding of the teleconsultation projects, improve construct validity and reliability [35, 43], and partially address both key informant and researcher bias issues [35].

### Data analysis

The transcribed interviews were analyzed and coded, based on the coding scheme presented in Table 4. Interviews relevant to a particular case (teleconsultation project) were first coded, and the coded interview segments across key informants for that given case were grouped together, analyzed, compared, and integrated in an iterative process. Each case was written up on its own in order to integrate the relevant interviews for each teleconsultation project into one document. This resulted in a more complete and coherent understanding of each individual project than would have been possible by analyzing each interview separately. Each case was then reanalyzed, recoded, and compared against the others. The use of computer-aided qualitative data analysis software enhanced coding reliability by making possible more consistent, frequent, and in-depth comparative analysis [44–46]. It also enhanced confidence in internal validity by more readily facilitating the constant comparison and pattern matching of the different coding values assigned first within and then between cases [35, 44].

**Table 4:** Coding scheme overview

I. GENERAL PROJECT BACKGROUND/PARTICIPANTS	B. Patient Demographics
A. Relationship -- HSC and Remote Site	C. TC Participants
Relationship -- Formal	TC Participants -- HSC
Relationship -- Personal	TC Participants -- Remote Site
*Prior Familiarity*	*Remote Site Participant Expertise*
*Trust*	*Remote Site Training*
B. HSC Specialty	
C. Physical/Plant Description of Remote Site	VI. TELCONSULTATION SESSIONS
Remote Site Resource Issues	A. TC Session Scheduling
	TC Frequency
II. HEALTH CARE DELIVERY PROBLEM	TC Session Length
A. Health Care Complexity	B. TC Session Description
Diagnosis	TC Session Process
Treatment	
Disease Quirks	VII. PROJECT IMPACT
	A. TC suitability -- What Could/ Not Be Done
III. PROJECT INITIATION (when/why started)	HSC Provider/Patient Connection -- Psychological Patient Acceptance
A. Date Project Started	B. TC Outcomes -- Examples
B. Project Startup	TC Outcomes -- (Before /After)
Initial Activities	TC Outcomes -- Failures
Barriers to Startup	TC Outcomes -- Readmittance
C. Need to Project	C. Access to Care -- Overall
	Access to Care -- Project Volume
IV. INFORMATION TECHNOLOGY (IT) CONFIGURATION	Access to Care -- TC Efficiency
A. IT Equipment	D. Cost of Care -- Overall
IT Location	Cost of Care -- Project Financing
IT Description	Cost of Care -- Reimbursement
B. User Perceptions	E. Quality of Care -- Overall
IT Training	Quality of Care -- Reputation
IT Ease-Of-Use	Quality of Care -- Referral Patterns
IT Reliability/Problems	Quality of Care -- Local Expertise
IT Sufficiency	
Local IT Support Availability/Expertise	VIII. PROJECT FUTURE
	A. Future -- Issues to Be Resolved
V. TELCONSULTATION (TC) PROJECT BACKGROUND	
A. TC Project Purpose	
Frequency of Activites (non-TC)	

Key informant interviews were used to identify the drivers influencing healthcare providers participating in teleconsultation projects, possible design steps to address these drivers, and the potential impact on remote site healthcare delivery. The generally accepted barriers to teleconsultation project sustainability, including reimbursement and technology, were first examined and found not to have explanatory power. Commonalities among teleconsultation projects that were sustained were then identified, as were commonalities among those projects that were discontinued or effectively dormant. Note that examples presented in the Results section usually involve only one key informant. In most cases (including those presented), multiple confirming comments from different key informants involved in that particular project, in addition to other forms of evidence identified in Table 1, were used to determine the findings.

## Results

### Key drivers influencing healthcare provider continued participation

The first research question asked: What are the significant drivers influencing healthcare providers’ continued participation in teleconsultation projects?

### Key driver: HSC specialists & remote site healthcare providers (RSHCPs) time constraints

HSC specialists and RSHCPs faced severe time constraints in general. Both sets of healthcare professionals worked in environments where they already had many other responsibilities that tended to place excessive demands on their time. Telemedicine added to these demands, yet was not considered to be, nor was it likely to become, a major priority or significant part of their job responsibilities. Healthcare providers might be able to tolerate these additional demands on their time in the short run – particularly if they were involved in one-off demonstration projects. However, as telemedicine moved to being primarily operational projects, these additional demands on their time likely significantly constrained their continued participation in such projects.

That these conditions held for the HSC specialists was illustrated by how little time was allocated to the actual teleconsultation sessions for each project. As shown in Table 2, none of the teleconsultation projects met on a formal basis more than once per week, and only Project HCV (at 4 hours total) and the school clinic project (at 2 hours and on an as-needed basis) averaged two or more hours per week. These limitations in time allocated to projects were primarily the result of HSC specialists’ time constraints. For example, in the burn unit project, teleconsultation sessions were scheduled for an hour every other week, because of HSC Y’s Burn Specialist’s time limitations.

This is not to say that only HSC specialists faced significant time constraints. For RSHCPs, time constraints were the result of their practicing in HSPAs and Medically Underserved Areas/Populations. This meant they were often shorthanded and had many additional responsibilities besides those related to teleconsultation projects. Both HSC specialists and RSHCPs severe time constraints considerably limited the time available for teleconsultation sessions, and there was a limit to the number of patients that could be seen during such sessions. As a result, this made the traditional healthcare delivery model of primary care providers transferring responsibility of care to specialists impractical in telemedicine. HSC Z’s DABC Psychiatrist stated:

*When they [the RSHCPs] think of the direct model service [where HSC specialist takes over the patient’s care], they quickly realize that they would—if I’m available let’s say 2 hours a week, if they gave me a caseload that I would manage then I would touch the lives of very few people, I mean with that length of time.*

### Key driver: remote site participant sense of professional isolation

Interestingly, it appears that RSHCPs sense of professional isolation trumped their time constraint concerns when it came to their continued participation in teleconsultation projects. RSHCPs sense of professional isolation in large part was the consequence of limited healthcare resources available at remote sites, and a patient base that was poor and at best covered by Medicaid or a similar government program–if they had any insurance at all. Project HCV Z4’s Physician Assistant exhibited how this made her feel when she stated: “It is so frustrating to work in an environment like this. You know, you don’t have a lot of resources.”

RSHCPs sense of professional isolation was further reinforced by how they perceived these limitations negatively impacting their ability to provide quality care. For example, patients’ economic status often prevented their traveling to an HSC to seek specialty care, and there were limitations on the effectiveness of communicating with specialists by telephone or facsimile. A primary care physician from Remote Site Y1’s multiple specialties project described the situation:

*Interviewer: How were some of these cases handled prior to telemedicine?*

*Physician: We just took a wild-ass guess. We did, because a lot of these folks can’t get up there [to the HSC]. You know, you can try calling, but it's not the same thing as looking at it, and so a lot of it was just…You just used your best judgment and went on.*

### Design steps to address participant drivers

The second research question asked: How can teleconsultation projects be designed to effectively and efficiently address these drivers? The findings indicated that the teleconsultation projects that were sustained over time had two affordable design characteristics in common, which addressed concerns about both time constraints and RSHCPs sense of professional isolation.

### Design for convenience by implementing technology and process solutions

The first design step required teleconsultation sessions be designed so that they were convenient for participants. This was often rather simple to accomplish by relatively straightforward technology and process design solutions. The technology solution involved physically locating telemedicine workstations where participating healthcare providers perceived them as being readily accessible—such as places where healthcare providers usually were during their normal course of activities. This might sound obvious, but it was not until the time of the later data collection period, when telemedicine workstation cost had fallen from more than $50,000 to just a few thousand dollars, that this was financially feasible. For example, HSC Y’s Oncologist had previously participated in a failed teleconsultation project that was discontinued in large part because it took up too much time for HSC specialists to travel to the inconveniently located telemedicine workstation. In contrast, the new oncology project had two workstations that were conveniently located at different clinics within the HSC. For RSHCPs, the actual location of the workstations was less of an issue given the usually small size of the healthcare facilities in which they worked.

What was more important to RSHCPs from a convenience standpoint could be addressed by process changes. This was achieved by scheduling the teleconsultation sessions in advance so that the participating healthcare providers felt the sessions were just another part of their normal work activities. Neither RSHCPs nor HSC specialists liked unscheduled, emergency teleconsultations because they considered them a significant burden that interrupted their already full schedules. This process solution was consistent with how teleconsultation projects actually were being utilized. Although the usefulness of telemedicine in the case of remote site emergencies was touted as a significant advantage of such projects, the reality was that the need for such emergency teleconsultation sessions rarely occurred. HSC Y’s IT professional responsible for setting up the technology for each teleconsultation session estimated, “We had two emergency consults last year and the rest of them were all scheduled; it’s really not a strain.” What RSHCPs generally faced were quasi-emergencies that required a consult with a specialist within a few days. It turned out that these consults could be scheduled one to three days beforehand, or dealt with during the regularly scheduled teleconsultation sessions. This enabled healthcare providers on both sides to schedule their day.

### Design projects to facilitate learning

The second design step required the repurposing of teleconsultation projects themselves so that they were designed to facilitate learning instead of directly providing healthcare services. It was unlikely there ever would be enough time allocated to teleconsultation sessions if a traditional healthcare delivery model where RSHCPs transferred responsibility for their patients to HSC specialists was utilized. Therefore, what was required was the creation of a new model of healthcare delivery where the underlying philosophy of teleconsultation projects shifted from providing healthcare to building local capacity.

This was accomplished by the teleconsultation sessions being designed to leverage HSC specialists’ expertise by empowering participating RSHCPs so that they could handle both more complex and a wider variety of healthcare problems. Project DABC’s Psychiatrist described what they did during their weekly 2-hour teleconsultation sessions and the impact it had:

*If I do case discussions [with RSHCPs], some of which can be very brief and some of them are these collaborative interviews which might be an hour, an hour-and-a-half, I’m touching an awful lot of patients and their families and other professionals. And so I’m just more useful to them [RSHCPs]. You know, sort of like they’re extending the utility input someone like me could provide.*

Likewise, Project ECDD’s Senior Communication Specialist gave an example of this when he explained what they were trying to do:

*I am trying something a little bit different because I do not want to be perceived as the expert on the other side of the TV set telling these people what to do…So we [ECDD Program Consultant] are doing this together so that we are really working to empower the service coordinators and the families to implement things within their everyday life, and that it is not a magic hands therapy technique to change these kids.*

This idea was supported by what the teleconsultation sessions were actually used for. RSHCPs almost never used the teleconsultation sessions for definitive diagnoses. Instead, they generally were used to assist RSHCPs in making sense of and thus better addressing complex or unusual problems. A RSHCP physician involved with multiple specialties project Y1 commented:

*You know, most of us can figure out what needs to have a procedure and which ones don’t. Most of its coming down to, you know, data management, reassurance, and that kind of thing. Very rarely do we not have any kind of idea at all of what is happening.*

Implementing the second design step required significant changes in terms of the process and content of the teleconsultation sessions themselves. First, the educational component was critical because empowerment could be achieved only by RSHCPs learning how to deal with more problems on their own. Z4’s Physician Assistant gave an example of how learning was facilitated in the HCV teleconsultation sessions:

*He [HSC HPV Specialist] makes the point to make every opportunity to learn [that] is possible. If somebody presents a patient and there’s any opportunity, he’ll say, “Okay, let’s stop here. I wanna explain why I‘m gonna tell you to do this.” And he’ll explain it and you leave feeling like I totally know this now.*

Second, this learning primarily had to be the type of learning that could not be gained by just reading textbooks. The same Physician Assistant stated:

*“In medicine, we’re always trained on patients because you can read all that you want but nothin’ is ever a classic case of whatever, you know. It’s always complicated by five other disease processes.”*

Instead, the learning had to be accomplished by RSHCPs actively participating and taking a leading role in patient care. Project DABC’s Psychiatrist explained:

*I work with you [RSHCPs] actively with the idea that your competence and confidence gradually increases over time and I play much more of a consultative secondary role. But that’s different from either if you assign me a caseload and I’m a direct provider of service, or we never interact directly with the patients that you see.*

Third, the teleconsultation sessions had to be a collaborative process between HSC specialists and RSHCPs, and between the different RSHCPs themselves. Often, this collaboration involved multidisciplinary teams from the HSC. For example, in Project HCV, HSC participants in the weekly teleconsultation sessions included two psychiatrists, a substance use disorder physician, an infectious diseases specialist, and the hepatitis C specialist himself.

Finally, to keep participating RSHCPs interested and continuing to participate, this learning process had to be almost continuous because of the constantly changing nature of the problems faced; otherwise one-off training would be sufficient. This appeared to hold regardless of whether RSHCPs were physicians, physician assistants, nurses, therapists, or developmental specialists. The Nurse at Project HCV Site Z2 stated, “With every presentation and every patient you have, you’re constantly learning. You’re constantly learning.” This belief was echoed by Project HCV Site Z1’s Physician when he stated:

*And the whole idea is that you learn quite a bit. If maybe ten people call in, and everybody is presenting a patient. By listening, I learn of a patient’s problem and what to do about it, you know?…So it’s like a continuous wheel for learning, you know what I mean?*

Indeed, Project HCV Site Z4’s Physician Assistant believed that the learning component was critical to keeping RSHCPs interested in participating in teleconsultation projects:

*(I)f that [learning] weren’t there, if he [HSC Hepatitis C Specialist] was just like, “Okay, do this. Okay, do this and they’ll be fine,” then I’d be like what the heck are we doing? I’m not learning anything. I think that learning component has to be there or people lose interest.*

The impact and importance of the teleconsultation project to Z4’s Physician Assistant was illustrated when she commented:

*To have that resource [telemedicine] and to feel like I’m actually doing something is just awesome. I need that kind of jobs aspect, you know what I mean?…I just feel like so connected to a bigger medical community that way…So yeah, I love it. If I wasn’t involved in this program, I would not stay at this job. No way.*

## Discussion

This research has examined the issue of teleconsultation project sustainability from the perspective of participating healthcare providers. However, their continued participation and project sustainability are not ends in and of themselves; rather, they are useful only if they improve healthcare delivery at remote sites. Implementing the design steps identified in this research (learning and convenience) has the potential to do so in a number of additional ways than just by providing limited increased access to HSC specialists.

The increased expertise of RSHCPs enabled them to handle more complex problems locally without the need to refer patients to HSC specialists. This made them better able to handle patients requiring follow-up care in general, and, in particular, the many challenges patients with chronic conditions face. This had the potential to reduce the cost of care because such patients are more likely to be compliant and thus cheaper to treat if keeping such appointments is easy and convenient. HSC specialists and RSHCPs time constraints concerns were further addressed because the ability of RSHCPs to handle more complex problems on their own resulted in fewer teleconsultation sessions.

Further, when teleconsultation sessions did occur, the evidence suggests that access to HSC specialists was increased in that they could see more patients in a given amount of time than they could see face-to-face at their clinics. For example, HSC Y Burn Specialist estimated he tended to see nine to ten patients per hour via telemedicine compared to five or six in a face-to-face setting. One reason for this was process-based, where the teleconsultation sessions required changes that shifted the workload away from HSC specialists. For example, in teleconsultation sessions, it was the patient who had to in effect change rooms, unlike face-to-face sessions where it was the specialist who had to move from patient to patient. An additional reason was that RSHCPs, by seeing patients on a regular basis and often being their primary care provider, were more familiar with the patients than whoever prepped them at the HSC clinic. Combined with their increased expertise, RSHCPs could better predict the relevant background information HSC specialists might or might not require, and this enabled RSHCPs to proactively provide such information without being asked.

In effect, RSHCPs were often required to take on a number of additional roles and responsibilities not necessary in a face-to-face setting. This highlighted the importance of trust in the relationships between HSC specialists and RSHCPs. HSC specialists had to trust the competency of RSHCPs, and RSHCPs had to be willing to take on new roles and trust that HSC specialists would be accepting of such new roles and input. Further, by combining HSC specialists’ expertise with RSHCPs’ local knowledge of the patient, teleconsultation projects had the potential to provide higher quality integrated care. For example, Project HCV had collected preliminary data showing that the outcomes for patients treated for hepatitis C via the teleconsultation project were as good if not better than the results of patients being treated at HSC Z only.

Teleconsultations sessions utilized as a means by which to exchange information were particularly useful when the information required was complex and needed to move the process from one stage to the next when addressing patient healthcare issues. The findings indicated that the most useful telecommunication consultations occurred when both sides of the teleconsultation project were learning from the exchange. This raised the level of consultation and enabled growth of all parties involved.

The findings indicated that the importance and effectiveness of designing for learning held regardless of whether participants were physicians or other healthcare professionals. Project ECDD‘s HSC participants were primarily non-physician specialists, yet their attitudes and beliefs about the philosophy of the teleconsultation projects and the importance of learning were consistent with those expressed by HSC physicians involved in other projects. Although only three of the teleconsultation projects (Project HCV’s Z1 and Z3 and Y4’s Oncology) involved physicians at remote sites, there appeared to be little or no difference between these and other RSHCPs in terms of the critical role continuous, collaborative learning played in their decision to continue to participate in teleconsultation projects. Rural areas also tend to have significant challenges in both attracting and retaining healthcare providers [47–49], and the learning aspect of teleconsultation projects can help overcome the sense of professional isolation that often contributes to RSHCPs leaving.

The findings also indicated that, when comparing individual teleconsultation projects, there was no evidence to suggest that severe time constraints were an even greater problem for those projects that were discontinued by the healthcare participants themselves (as opposed to the telemedicine network); rather, the healthcare providers involved did not perceive the teleconsultation projects as being of sufficient value to continue to participate in relative to the time commitment required.

Although outside the scope of this article, it should be noted that, contrary to what was probably the most common reason given in the literature [4, 6, 14], limited reimbursement was not perceived as a major barrier to telemedicine project sustainability. For the HSCs, there were a number of possible explanations for this. First, the amount of time that individual participating HSC specialists allocated to non-specialty teleconsultation projects was quite limited and averaged approximately one session per month. Second, in the case of the specialty teleconsultation projects involving conditions with long-term treatment regimens or follow-up, HSCs often were reimbursed on a global fee basis—making teleconsultation session reimbursement a moot point. Third, many of the teleconsultation sessions involved indigent care, where HSC specialists were not going to be reimbursed whether the patient was seen via telemedicine or in the clinic. Moreover, the HSCs studied had not developed the administrative processes necessary to file reimbursement claims for eligible teleconsultation sessions. Finally, many of the teleconsultation project sessions were not eligible for remote site reimbursement because the patient was not present during the teleconsultation sessions themselves.

### Contributions to research and practice

This research makes significant contributions to research and practice by making researchers, policy makers, and participating organizations aware of what influences healthcare providers’ continued participation in teleconsultation projects, and by providing implementable and affordable design steps to address these influencing factors. It also provides an alternative perspective on designing, implementing, and evaluating teleconsultation projects to facilitate their sustainability.

The findings indicated that the challenges of provider time constraints and remote participant professional isolation can be effectively and efficiently addressed by designing the teleconsultation projects for convenience and to facilitate learning. Mechanisms by which RSHCPs were empowered through the leveraging of HSC specialists expertise when so little time was actually dedicated to teleconsultation sessions themselves were identified. This research also further explains why teleconsultations appear to be especially relevant to effectively managing chronic conditions and those with long treatment regimens by facilitating and thus increasing the likelihood of patient compliance over the long term.

This research is consistent with and helps explain why teleconsultation projects sustained over time were collaborative in nature and included the active participation of all the healthcare providers involved. This study deepens our understanding of why interpersonal trust is a necessary precondition for telemedicine projects to have a positive impact on remote site healthcare delivery [39]. Active, continuous learning is a collaborative process that requires not only interpersonal trust of the other parties but trust that the technology is reliable and suitable for the demands learning places on it, and trust that the processes that facilitate the teleconsultation sessions themselves are necessary and properly carried out.

This research also provides a counter argument to the popularly-held belief that technology requirements for effective teleconsultation must be quite advanced because they must replicate the face-to-face experience [1, 4, 38, 50]. Instead, it supports and helps explain why other studies have found that technology challenges related to teleconsultation projects deal with the technology being too complex and having more functionality than needed, and, in some cases, not having the relatively basic functionality healthcare provider participants wanted [38, 51].

### Limitations

This research is not without its limitations. First, even though drawing on data collected at two different points in time, this study was not actually multiperiod because much of the data included cases that were not active at the time of the first study. However, it can be argued that in some ways this further strengthens the findings presented because inferences were able to be drawn from data about projects that were relatively inactive or not sustained, and these inferences could be compared against the characteristics of those teleconsultation projects that were sustained over time. It is argued the timing of the two data collection periods were appropriate and enabled the collection of the necessary data. While there are many reasons for this, a key reason was that most telemedicine projects at the time of the first data collection period started as pilot studies or proof of concept, while those from the second data collection period occurred after the efficacy and efficiency of telemedicine for many clinical activities had been established and the deployed teleconsultation projects were now being done as part of organizations’ ongoing operations.

Second, this research involved only teleconsultation projects located in the United States, which has its own characteristics in terms of healthcare providers, payers, and regulations which may not hold in other parts of the world. While these findings are consistent with and extend prior studies done in Australia, whose healthcare system differs significantly from that of the United States [28], this research needs to be replicated in additional countries with differing healthcare systems.

Third, while the sample size was limited, it is argued that the diversity in the types of healthcare activities practiced, the professional qualifications of healthcare providers involved, and population size, location, and remoteness of the sites themselves makes this an appropriate sample. The results between those teleconsultation projects located in areas designated metropolitan and those in nonmetropolitan areas exhibited no meaningful difference. The majority of remote sites in this study were located in nonmetropolitan areas were in effect rural, and rural areas tend to face healthcare challenges that are similar to or in some cases more pronounced than urban areas because rural populations tend to be poorer, older, and have higher rates of certain chronic diseases [4, 27, 52].

### Future research

In addition to addressing the limitations discussed above, future research needs to examine the effect of widespread deployment of electronic health records (EHRs) shared by both remote sites and HSCs. At the time of the second data collection period, only Primary Care Project Y5 had integrated the use of EHRs into its teleconsultation sessions (HSC Y had integrated the state’s Department of Corrections’ EHR into their correctional facility telemedicine projects). None of the other projects had done so. Most had not even integrated the ability to receive laboratory reports in a format other than paper or facsimile. As a result, RSHCPs believed administrative burdens related to their continued participation in teleconsultation projects presented additional time constraints. Future research is needed to determine the extent to which integrated EHRs can help address the increased administrative overhead RSHCPs often face as a result of their continued participation. It is also needed to better understand whether teleconsultation projects with integrated EHRs can further improve the quality of care by enabling patients to receive more integrated care. This could be especially important as healthcare moves from episodic to preventative care.

Future research is needed to address whether or not healthcare providers require certain characteristics, and whether those characteristics can be determined by professional qualifications or are individually-based. Teleconsultation project participants require a certain level of qualifications to take advantage of the learning aspects, but the results from this study suggest it is individual characteristics and not professional qualifications that matter more. Future research is needed to determine whether it is the ability and willingness of the participants, given a certain level of training not specific to physicians, to learn and assume new roles that is more important to their continued participation than is the formal professional qualifications RSHCPs hold. Furthermore, given the status differentials between the project participants, a better understanding of the social processes and power dynamics involved in teleconsultation projects might also be needed.

## Conclusion

This research builds on prior research on telemedicine service sustainability by examining successfully implemented telemedicine projects from healthcare provider participants’ perspective. Key drivers influencing healthcare providers’ continued participation in such projects and two design steps that can be taken have been identified. The most important of these steps is to design the consultation process as a learning process. We show how taking these design steps can impact healthcare delivery in medically underserved areas.

### Ethics approval and consent to participate

The University of Texas at Austin’s Institutional Review Board approved the study involving the first data collection period, and the University of Denver’s Institutional Review Board approved the study involving the second data collection period.

### Availability of data and materials

Signed confidentiality agreements prevent us from sharing the data.

## Abbreviations

DABC: drug abuse and behavioral counseling project
ECDD: early childhood developmental disabilities project
HCV: hepatitis C virus project
HPSA: health professional shortage area
HSC: health sciences center
IT: information technology
RSHCP: remote site healthcare provider
